# NPA Hierarchy and Extremal Criterion in the Simplest Bell Scenario

**DOI:** 10.3390/e27020182

**Published:** 2025-02-09

**Authors:** Satoshi Ishizaka

**Affiliations:** Graduate School of Advanced Sciences and Engineering, Hiroshima University, 1-7-1 Kagamiyama, Higashi-Hiroshima 739-8521, Japan; isizaka@hiroshima-u.ac.jp

**Keywords:** Bell inequality, simplest Bell scenario, NPA hierarchy, 03.65.Ud, 03.65.Ta, 03.67.Hk, 03.67.Dd

## Abstract

It is difficult to establish an analytical criterion to identify the boundaries of quantum correlations, even for the simplest Bell scenario. Here, we briefly reviewed the plausible analytical criterion, and we found a way to confirm the extremal conditions from another direction. For that purpose, we analyzed the Navascués-Pironio-Acín (NPA) hierarchy to study the algebraic structure and found that the problem could not be simplified using the 1+AB level. However, considering the plausible criterion, the 1+AB and second levels for correlations were equal, and the extremal condition in the simplest Bell scenario was replaced by that in the 1+AB level. Thus, the correctness of the plausible criterion was verified, and the results demonstrated that the plausible criterion held, thereby explaining its simplicity. It seemed plausible, but now it becomes more certain.

## 1. Introduction

In 1964, Bell showed that nonlocal correlations predicted by quantum mechanics were inconsistent with local realism [1]. Although the non-local correlations did not contradict the no signaling principle, the strength of the quantum correlations was more restricted than that allowed by the no-signaling principle [2,3]. Since then, fundamental principles have been investigated to address this discrepancy [4,5,6]. Furthermore, the origin of fundamental principles, such as uncertainty and nonlocality, has been investigated [7,8]. However, in these studies, clarifying the boundaries of quantum correlations was difficult. Indeed, an analytical criterion for identifying the boundaries has not yet been established, even for the simplest Bell scenario.

In the simplest Bell scenario, Tsirelson showed that the Bell inequality of the Clauser–Horne–Shimony–Holt (CHSH) type [9] is violated up to $2\sqrt{2}$ by quantum correlations [2]. The correlation that attains the Tsirelson bound is an extremal point in a convex set of quantum correlations. When the marginal probabilities of obtaining the measurement results are unbiased (zero-marginal case), the boundaries are identified using the analytical criterion Tsirelson–Landau–Masanes (TLM) [10,11,12]. Recent studies have shown that there are many characteristics of the boundaries in a quantum set [13].

In the general case where the marginals may be biased (full-marginal case), obtaining the analytical criterion is a long-standing open problem. In fact, only a few examples of analytical solutions are known [8,14,15,16,17]. The geometry of a quantum set exhibits rich counterintuitive features [17]. We propose a plausible analytical criterion to identify all extremal points (a point is an extremal point if and only if the equalities Equations (12) and (13) are simultaneously satisfied) [18]. Recently, some quantum states have been discussed, and it has been shown that the plausible criterion is a strong indication, which, however, remains unproven [19].

The plausible criterion proposed in [18] is simpler than expected. Therefore, we analyze the Navascués–Pironio-Acín (NPA) hierarchy [20,21,22] to study the algebraic structure. Specifically, the sets of correlations are denoted as $Q^{\left(1\right)}$, $Q^{\left(1+AB\right)}$, $Q^{\left(2\right)}$, ⋯ (it is also called the correlation levels between the first, 1+AB, second, and others correspondingly). By definition, the inclusion relation generally is $Q^{\left(1\right)}⊇Q^{\left(1+AB\right)}⊇Q^{\left(2\right)}⊇\cdots ⊇Q$, where *Q* is the set of correlations in the simplest Bell scenario and all the extremal points are conjectured by the plausible criterion. If a phenomenon (in a Bell inequality) is explained up to the 1+AB level, then $Q^{\left(1\right)}⊇Q^{\left(1+AB\right)}=Q^{\left(2\right)}=\cdots =Q$ holds true. This becomes clear upon examining the inclusion relationship between the 1+AB and second levels (at this time the following condition is automatically satisfied by a rank loop: $Q^{\left(2\right)}=Q^{\left(3\right)}=\cdots$ [21]). In the NPA analysis, the simplicity of the plausible criterion is persuasive, especially when the 1+AB and second levels are equal (just as the TLM criterion is explained by the first and second levels). Otherwise, in this study, we say nothing exceptional about the 1+AB level.

In this paper, we briefly review the plausible criterion for convenience, and we find a way to confirm the extremal conditions from another direction. For that purpose, we analyze the NPA hierarchy to study the algebraic structure, and the results show that the 1+AB level is not exceptional. After all, the problem cannot be simplified using the 1+AB level. However, as far as the plausible criterion is concerned, the 1+AB and second levels for correlations are equal, and the extremal condition in the simplest Bell scenario is replaced by that in the 1+AB level. Thus, the correctness of the plausible criterion is clarified, and the results demonstrate that it holds true, which explains its simplicity.

## 2. Plausible Criterion

We now clarify the plausible criterion obtained in the two parity Alice and Bob system; the projective measurements of Rank 1 are performed in a two-qubit entangled state. Specifically, without loss of generality, the observables are

(1) $$A_{x}=\cos\theta_{x}^{A}\sigma_{1}+\sin\theta_{x}^{A}\sigma_{3},\;B_{y}=\cos\theta_{y}^{B}\sigma_{1}+\sin\theta_{y}^{B}\sigma_{3},$$

where $\left(\sigma_{1},\sigma_{2},\sigma_{3}\right)$ are the Pauli matrices, and the two-qubit entangled state is

(2) |ψ〉=cosχ|00〉+sinχ|11〉(0<χ≤π/4).

Based on this parameterization, we obtain

(3) $$\begin{array}{ccc} \langle A_{x}B_{y}\rangle & = & \sin\theta_{x}^{A}\sin\theta_{y}^{B}+\cos\theta_{x}^{A}\cos\theta_{y}^{B}\sin2\chi , \end{array}$$

(4) $$\begin{array}{c} \;\langle A_{x}\rangle =\sin\theta_{x}^{A}\cos2\chi ,\;\langle B_{y}\rangle =\sin\theta_{y}^{B}\cos2\chi . \end{array}$$

The plausible criterion is classified into two categories. The first is based on the limitations of entangled states. Based on the analysis presented in Ref. [18], the usage of the entanglements are specified by the eight parameters $S_{xy}^{\pm }$, which obey the following correlations:

(5) $$\begin{array}{ccc} S_{xy}^{\pm } & \equiv & \frac{1}{2}\left[J_{xy}\pm \sqrt{J_{xy}^{2}-4K_{xy}^{2}}\right], \end{array}$$

(6) $$\begin{array}{ccc} J_{xy} & \equiv & {\langle A_{x}B_{y}\rangle }^{2}-{\langle A_{x}\rangle }^{2}-{\langle B_{y}\rangle }^{2}+1, \end{array}$$

(7) $$\begin{array}{ccc} K_{xy} & \equiv & \langle A_{x}B_{y}\rangle -\langle A_{x}\rangle \langle B_{y}\rangle . \end{array}$$

Here, for each *x* and *y*, $\sin2\chi \;=\;S_{xy}^{+}$ or $\sin2\chi \;=\;S_{xy}^{-}$ always holds true. If a two-qubit realization cannot be guaranteed for Equation (3), the condition

(8) $$\prod_{xy}\left\{\left(1-S_{xy}^{+}\right)\langle A_{x}B_{y}\rangle -\langle A_{x}\rangle \langle B_{y}\rangle \right\}\geq 0,$$

is necessary; see the Supplemental Material of Ref. [18]. The maximum use of the entanglements is $S_{00}^{+}\;=\;S_{01}^{+}\;=\;S_{10}^{+}\;=\;S_{11}^{+}$.

The second category is the cryptographic quantum bound [23]. The TLM inequality becomes an equality whose correlation is divided guessing probability. The scaled TLM inequality is expressed as

(9) $$\begin{array}{ccc} \left|{\tilde{C}}_{00}{\tilde{C}}_{01}-{\tilde{C}}_{10}{\tilde{C}}_{11}\right| & \leq & {\left(1-{\tilde{C}}_{00}^{2}\right)}^{1/2}{\left(1-{\tilde{C}}_{01}^{2}\right)}^{1/2}\\ & & +{\left(1-{\tilde{C}}_{10}^{2}\right)}^{1/2}{\left(1-{\tilde{C}}_{11}^{2}\right)}^{1/2} \end{array}$$

where ${\tilde{C}}_{xy}\;=\;\langle A_{x}B_{y}\rangle /D_{x}^{B}$ and ${\tilde{C}}_{xy}\;=\;\langle A_{x}B_{y}\rangle /D_{y}^{A}$. The guessing probabilities are as follows:

(10) $$\begin{array}{ccc} D_{x}^{B} & \;\;\;=\;\;\; & \sqrt{\sin^{2}\theta_{x}^{A}+\cos^{2}\theta_{x}^{A}\sin^{2}2\chi }=\mathrm{tr}\left|\rho_{1|x}^{B}\;-\;\rho_{-1|x}^{B}\right|, \end{array}$$

(11) $$\begin{array}{ccc} D_{y}^{A} & \;\;\;=\;\;\; & \sqrt{\sin^{2}\theta_{y}^{B}+\cos^{2}\theta_{y}^{B}\sin^{2}2\chi }=\mathrm{tr}\left|\rho_{1|y}^{A}\;-\;\rho_{-1|y}^{A}\right|, \end{array}$$

where $\rho_{a|x}^{B}\;=\;{\mathrm{tr}}_{A}\frac{I+aA_{x}}{2}\left|\psi \rangle \langle \psi \right|$ and $\rho_{b|y}^{A}\;=\;{\mathrm{tr}}_{B}\frac{I+bB_{y}}{2}\left|\psi \rangle \langle \psi \right|$. The NPA inequality is also extended to include $D_{x}^{B}$ and $D_{y}^{A}$ (see Equation (B4) in Ref. [23]). However, it is not used because full-rank is necessary and a two-qubit pure state does not satisfy the equality. Therefore, it is not an appropriate extremal condition.

Thus, our plausible criterion is as follows:

(12) $$\begin{array}{c} \;S_{00}^{+}=S_{01}^{+}=S_{10}^{+}=S_{11}^{+}, \end{array}$$

(13) $$\begin{array}{ccc} \left|{\tilde{C}}_{00}{\tilde{C}}_{01}-{\tilde{C}}_{10}{\tilde{C}}_{11}\right| & \;\;=\;\; & {\left(1-{\tilde{C}}_{00}^{2}\right)}^{1/2}{\left(1-{\tilde{C}}_{01}^{2}\right)}^{1/2}\\ & & +{\left(1-{\tilde{C}}_{10}^{2}\right)}^{1/2}{\left(1-{\tilde{C}}_{11}^{2}\right)}^{1/2}. \end{array}$$

A point is an extremal point if and only if the five equalities are simultaneously satisfied. The scaled TLM condition must be satisfied in both ${\tilde{C}}_{xy}\;=\;\langle A_{x}B_{y}\rangle /D_{x}^{B}$ and ${\tilde{C}}_{xy}\;=\;\langle A_{x}B_{y}\rangle /D_{y}^{A}$, and there are two equalities.

## 3. NPA Hierarchy and Simplest Bell Scenario

We now analyze the NPA hierarchy and use explicit examples to determine whether the 1+AB level is exceptional in the simplest Bell scenario. Table 1 lists the correlations of the quantum bounds ${\mathrm{QB}}_{2}^{\left(8\right)}$ and ${\mathrm{QB}}_{3}^{\left(8\right)}$, which are obtained using the quantifier elimination algorithm [16]. ${\mathrm{QB}}_{3}^{\left(8\right)}$ is discussed in relation to the plausible criterion [19]. In the table, the measures $x\langle A_{0}\rangle +\langle A_{0}B_{0}\rangle +\langle A_{1}B_{0}\rangle +\langle A_{0}B_{1}\rangle -\langle A_{1}B_{1}\rangle$ for ${\mathrm{QB}}_{2}^{\left(8\right)}$ and $x\langle A_{0}\rangle +x\langle A_{1}\rangle -x\langle B_{0}\rangle +\langle A_{0}B_{0}\rangle +\langle A_{1}B_{0}\rangle +\langle A_{0}B_{1}\rangle -\langle A_{1}B_{1}\rangle$ for ${\mathrm{QB}}_{3}^{\left(8\right)}$ are maximized, and the quantum values (theoretical values) are provided in the figure caption [16]. The calculated NPA 1+AB and 2nd levels are also listed, where a highly accurate multiple-precision semidefinite programming solver is used [24], and the same measures as the quantum values are maximized. In ${\mathrm{QB}}_{2}^{\left(8\right)}$, the 1+AB calculations are consistent with the quantum values (within the limits of numerical accuracy). In ${\mathrm{QB}}_{3}^{\left(8\right)}$, the 1+AB calculations deviate from the quantum values of approximately 0.8≤x≤1.0. Here, the endpoint x=1.0 is the classical regime according to the TLM criterion, but is influenced by the quantum regime with instability. Specifically, the deviation rapidly converges and remains within x=0.698 (figure is not shown); x≤0.7 seems accurate throughout corresponding to the non-deviate correlation. Thus, we conclude that deviate and non-deviate correlations coexist, and that there is nothing exceptional about the 1+AB level. The certificate is ${\mathrm{QB}}_{3}^{\left(8\right)}$ with 0.698≤x≤1.

**Figure 1:**
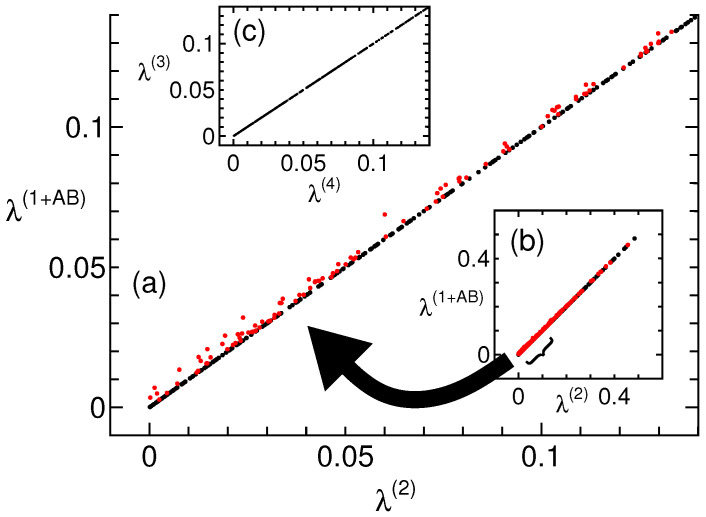
Maximum of λ, where Γ−λ≥0 at NPA 1 + *AB* level (vertical axis) and 2nd level (horizontal axis), obtained using the semidefinite programming solver [24]. (**a**) Plotted area near the origin and (**b**) full scale. The deviations from the straight line are indicated in red. (**c**) With data at 3rd (vertical) and 4th (horizontal) levels, for the levels equal to or greater than this, the two data are on a straight line. The number of data points are 500 to distinguish each data point.

Another semidefinite program demonstrated an inclusion relationship between the NPA 1+AB and second levels. It determines the maximum of λ such that Γ−λI≥0 (Γ−λI is nonlocal), where Γ is the certificate matrix [21]. Here, random initial points $\left\{\langle A_{x}B_{y}\rangle ,\langle A_{x}\rangle ,\langle B_{y}\rangle \right\}$, which are included in Γ, are common for the 1+AB and second levels. However, the maximum λ calculated using the 1+AB and second levels denotes $\lambda^{\left(1+AB\right)}$ and $\lambda^{\left(2\right)}$, respectively. Figure 1a,b shows the results of $\lambda^{\left(1+AB\right)}$ (*y*-axis) and $\lambda^{\left(2\right)}$ (*x*-axis). Because $Q^{\left(1+AB\right)}⊇Q^{\left(2\right)}$, $\lambda^{\left(1+AB\right)}$ is the same as $\lambda^{\left(2\right)}$ or shifted up. When $\lambda^{\left(1+AB\right)}$ and $\lambda^{\left(2\right)}$ are on a straight line, the 1+AB and second levels are always equal, and the correlations can be explained up to the 1+AB level. However, in the figure, approximately 30% of the data points deviate from the straight line (indicated in red), and deviated and non-deviated correlations coexist. Similarly, nothing exceptional is observed about the 1+AB level. How many orders of magnitude do we need to consider? Figure 1c shows the data of $\lambda^{\left(3\right)}$ (*y*-axis) and $\lambda^{\left(4\right)}$ (*x*-axis). At the levels equal to or greater than this, the two data are on a straight line, and two levels such as $\lambda^{\left(3\right)}$ and $\lambda^{\left(4\right)}$ are always equal. Thus, the second level is not enough (figure is not shown), and the third level is required.

**Figure 2:**
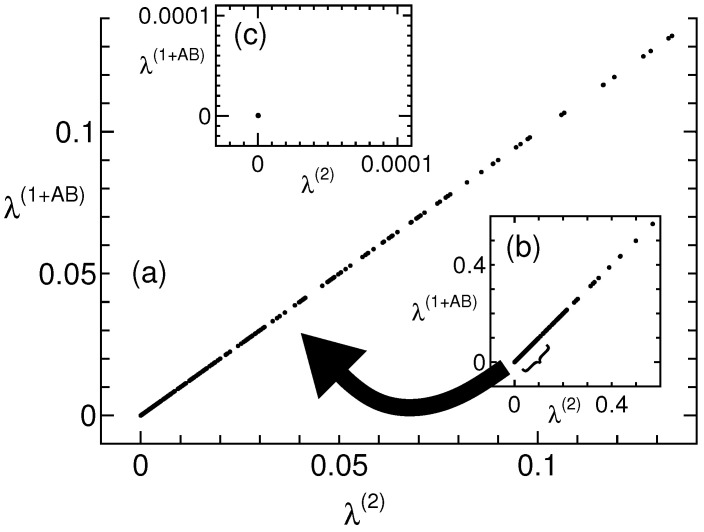
Two-qubit realizations and Equation (12) are randomly specified as initial points. These graphs are similar to Figure 1; however, they are selected by initial points. (**a**) Plots showing the area near the origin and (**b**) full scale. All the data points do not deviate from the straight line, and the NPA 1+AB and 2nd levels are always equal. (**c**) The plausible criteria (Equations (12) and (13)) are fully specified, and all the data points converge to a single point (0,0).

Two maximization problems exist: maximizing the quantum value (which depends on the state to be maximized) and maximizing λ such that Γ−λI≥0. Both yield the same results, and no exceptional results are observed for the 1+AB level. Thus, using the 1+AB level is unlikely to simplify the problem. However, maximizing λ such that Γ−λI≥0 is an interesting feature, which is revealed by selecting a fixed initial point in Figure 1. Specifically, two-qubit realizations are randomly specified as the initial points (pure states Equations (2)–(4) are specified considering Equation (8)). Thus, mixed states are exclusively removed; however, this does not change significantly from Figure 1. Thus, Equation (12) is additionally specified as the initial points. Figure 2a shows the results where none of the data points deviate from the straight line. Therefore, because it is a pure state and satisfies Equation (12), the 1+AB and second levels are equal. This will further equalize the correlations of the simplest Bell scenario, because a rank loop is established at the third level, as shown in Figure 1c, but for these initial points, the 1+AB and third levels are equal (figure is not shown). (Because ${\mathrm{QB}}_{3}^{\left(8\right)}$ is a pure state and satisfies Equation (12), the 1+AB and second levels are equal (${\mathrm{QB}}_{3}^{\left(8\right)}$ satisfies Equation (13) [19], because ${\mathrm{QB}}_{3}^{\left(8\right)}$ is a solution to the plausible criterion). However, in ${\mathrm{QB}}_{3}^{\left(8\right)}$ in Table 1, these levels are unequal, apparently because what is maximized is obviously different. Thus, even the relationship between the correlations varies depending on what is maximized.) Thus, when it is a pure state and Equation (12) is satisfied, the 1+AB and second levels are equal to those in the simplest Bell scenario.

We now consider what happens if the remaining plausible criteria are added to Equation (13). Figure 2c shows the results where all the data points ($\lambda^{\left(2\right)}$,$\lambda^{\left(1+AB\right)}$) converge to a single point (0,0). This convergence is explained as follows. First, the 1+AB and second levels are equal to the correlations of the simplest Bell scenario because they satisfy Equation (12). Consequently, the extremal condition in the simplest Bell scenario is replaced by that in the 1+AB level. Second, because $\lambda^{\left(1+AB\right)}$ and $\lambda^{\left(2\right)}$ are already optimal solutions of Γ−λI≥0, we obtain ($\lambda^{\left(2\right)}$,$\lambda^{\left(1+AB\right)}$) = (0,0). Therefore, after slight consideration, we find that the plausible criterion must be extremal points of the 1+AB level to ensure that ($\lambda^{\left(2\right)}$,$\lambda^{\left(1+AB\right)}$) converges to (0,0). Conversely, if the plausible criterion is incorrect and not a flat boundary point, convergence to (0,0) does not occur. Thus, the correctness of the plausible criterion is verified, although we are unsure of the existence of unnecessary flat boundary points. The convergence to a single point proves that the plausible criterion holds. Furthermore, the plausible criterion is simple because the simplest Bell scenario is explained by the 1+AB level. However, the method described is suitable for obtaining predictions; if predictions cannot be obtained, the method is unsuitable. In such cases, the Monte Carlo method may be used. However, an event is unlikely to survive hundreds of thousands of Monte Carlo trials. Our calculations do not reveal any exceptions to Equations (12) and (13) [18].

## 4. Summary

In summary, we analyzed the NPA hierarchy to study the algebraic structure in the simplest Bell scenario. The result is that the problem could not be simplified using the 1+AB level. However, when the problem is limited to the plausible criterion, the 1+AB and second levels for correlations are equal, and the extremal condition in the simplest Bell scenario is replaced by that in the 1+AB level. Although the existence of unnecessary flat boundary points remains unknown, the correctness of the plausible criterion is ascertained, and the results numerically demonstrate that the plausible criterion becomes more certain. Furthermore, this explains the simplicity of the plausible criterion.

## Figures and Tables

**Table 1:** Correlations of ${\mathrm{QB}}_{2}^{\left(8\right)}$ and ${\mathrm{QB}}_{3}^{\left(8\right)}$, obtained using the quantifier elimination algorithm in Ref. [16]. The superscript (8) denotes a fully-marginal 8D case to distinguish the zero-marginal 4D case. The quantum values (theoretical values) for ${\mathrm{QB}}_{2}^{\left(8\right)}$ are $\sqrt{2x^{2}+8}$ and, for ${\mathrm{QB}}_{3}^{\left(8\right)}$, are $\frac{\sqrt{\left(2-x^{2}\right)\left(4-3x^{2}\right)}-x^{2}}{1-x^{2}}$ (x≤1) and x+2 (1≤x≤2), respectively. The calculated NPA 1+AB and 2nd levels are also listed.

x	${\mathrm{QB}}_{2}^{\left(8\right)}$	1+AB	2nd
0.0	2.82842712474619	2.82842712474619	2.82842712474619
0.4	2.88444102037119	2.88444102037119	2.88444102037119
0.8	3.04630924234556	3.04630924234556	3.04630924234556
1.2	3.29848450049413	3.29848450049413	3.29848450049413
1.6	3.62215405525497	3.62215405525497	3.62215405525497
2.0	4.00000000000000	4.00000000000000	4.00000000000000
**x**	${\mathrm{QB}}_{3}^{\left(8\right)}$	1+AB	**2nd**
0.0	2.82842712474619	2.82842712474619	2.82842712474619
0.2	2.83091685885720	2.83091685885720	2.83091685885720
0.4	2.83923308963559	2.83923308963559	2.83923308963559
0.6	2.85676984164748	2.85676984164748	2.85676984164748
0.8	2.89417689813970	2.90075597099059	2.89417689813970
1.0	3.00000000000000	3.01789221335227	3.00737232088269
1.2	3.20000000000000	3.20000000000000	3.20000000000000
1.4	3.40000000000000	3.40000000000000	3.40000000000000
1.6	3.60000000000000	3.60000000000000	3.60000000000000
1.8	3.80000000000000	3.80000000000000	3.80000000000000
2.0	4.00000000000000	4.00000000000000	4.00000000000000

## Data Availability

The original contributions presented in this study are included in the article. Further inquiries can be directed to the author.
